# The ICON Trauma Study: the impact of the COVID-19 lockdown on major trauma workload in the UK

**DOI:** 10.1007/s00068-020-01593-w

**Published:** 2021-02-09

**Authors:** Alfred Adiamah, Amari Thompson, Christopher Lewis-Lloyd, Edward Dickson, Lauren Blackburn, Nick Moody, Sunil Gida, Angelo La Valle, John-Joe Reilly, John Saunders, Adam Brooks, The ICON Trauma Study Group, Alfred Adiamah, Fady Anis, Lauren Blackburn, Hilary Brewer, Adam Brooks, Rachel Brailsford, Shannon Boardman, Amanjeet Dahaley, Edward Dickson, Zoe Draper, Ramzi Freij, Wendy Gaskin, Sunil Gida, Lauren Hutchinson, Jamaall Jackman, Audrey Kapeleris, Amanda Koh, Christopher Lamb, Christopher Lewis-Lloyd, Angelo La Valle, Rachel Lee, Shane McSweeny, Yasar Nassif, Alex Navarro, Rory O’Connor, Ciara O’Sullivan, Olamide Oyende, John-Joe Reilly, Sharon Sanderson, John Saunders, Amari Thompson, Elena Theophilidou, Sandeep Walsh, Robert Winter

**Affiliations:** East Midlands Major Trauma Centre, Queen’s Medical Centre, Nottingham University Hospitals NHS Trust and University of Nottingham, Nottingham, NG7 2UH UK

**Keywords:** Major trauma, Covid-19, Injury severity, Mortality, ICON-TRAUMA

## Abstract

**Background:**

The global pandemic caused by SARS-CoV-2 has impacted population health and care delivery worldwide. As information emerges regarding the impact of “lockdown measures” and changes to clinical practice worldwide; there is no comparative information emerging from the United Kingdom with regard to major trauma.

**Methods:**

This observational study from a UK Major Trauma Centre matched a cohort of patients admitted during a 10-week period of the SARS-CoV-2-pandemic (09/03/2020–18/05/2020) to a historical cohort of patients admitted during a similar time period in 2019 (11/03/2019–20/05/2019). Differences in demographics, Clinical Frailty Scale, SARS-CoV-2 status, mechanism of injury and injury severity were compared using Fisher’s exact and Chi-squared tests. Univariable and multivariable logistic regression analyses examined the associated factors that predicted 30-days mortality.

**Results:**

A total of 642 patients were included, with 405 in the 2019 and 237 in the 2020 cohorts, respectively. 4/237(1.69%) of patients in the 2020 cohort tested positive for SARS-CoV-2. There was a 41.5% decrease in the number of trauma admissions in 2020. This cohort was older (median 46 vs 40 years), had more comorbidities and were frail (*p* < 0.0015). There was a significant difference in mechanism of injury with a decrease in vehicle related trauma, but an increase in falls. There was a twofold increased risk of mortality in the 2020 cohort which in adjusted multivariable models, was explained by injury severity and frailty. A positive SARS-CoV-2 status was not significantly associated with increased mortality when adjusted for other variables.

**Conclusion:**

Patients admitted during the COVID-19 pandemic were older, frailer, more co-morbid and had an associated increased risk of mortality.

## Introduction

In response to the global pandemic caused by SARS-CoV-2, the government of the United Kingdom (UK), in line with World Health Organisation advice [1], implemented a period of lockdown in an attempt to reduce the rate of transmission of the virus. These “lockdown measures” came into effect on the 23rd March 2020 [2] and led to unprecedented closures of social venues, public houses, bars and non-essential services, as well as a significant reduction in road usage. Whilst data regarding the effect of the pandemic and associated lockdown measures on orthopaedic injuries continues to emerge [3, 4], there is not much data regarding major trauma in the UK. Despite this, the seasonal variability in trauma admissions is well documented [5–7]. This has been attributed to decreased trauma incidents during the Easter and Christmas holidays and an increased pattern during Summer, school holidays and national bank holidays [5–7]. Whether this pattern will be replicated during a UK national lockdown; and the effect on the rate, type and severity of trauma presentations, is unclear. Earlier studies during the global pandemic reported significant decreases in the emergency medical [8] and surgical workload [9]. Anecdotal evidence from Italy suggested a fall in the number of emergency trauma admissions but an increase in injury severity [10]. More recent work from Spain and the United States documented a downward trend of emergency trauma admissions in their specific populations [11, 12].

The National Health Service (NHS) of the UK guidance on the management of trauma patients during the pandemic suggested delayed and non-operative management of injuries where possible [13]. The reconfiguration and redistribution of workforce and resources, to ensure hospital capacity was available for the predicted surge in patient admissions with SARS-CoV-2, may have also had an impact on the delivery of major trauma care. The ICON Trauma study [14] was designed to understand the impact of imposition of lockdown on the burden of trauma admissions in the UK. This will inform resource and workforce planning during the ongoing pandemic, particularly in anticipation of a second peak during winter.

## Methods

This retrospective observational study was undertaken at the East Midlands Major Trauma Centre, Nottingham University Hospitals NHS Trust which has a catchment area of approximately 3.8 million people. In accordance with NHS England directives, this Major Trauma Centre receives major injured patients directly from point of injury where travel times allow or following rapid stabilisation and transfer from a Trauma Unit with predetermined local protocols in place. On average, the team assesses and treats 40 major trauma patients each week. For the purposes of this study, a 10-week period that included the 2-weeks before and the 2 weeks after the first UK Nationwide lockdown was compared to a matched 10-week period in 2019, to account for the known seasonal variability in major trauma admissions.

### Definition

Major trauma was defined using the UK NICE guidelines definition which is accepted by all the UK national trauma centres as an injury or combination of injuries that are life-threatening and could be life changing because it may result in long-term disability [15].

### Inclusion

All consecutive trauma patients admitted during the 10-week period between 9th March and 18th May 2020, which coincides with the 2-week period before, during and the 2 weeks after the UK lockdown formed the 2020 cohort. Similarly, consecutive patients admitted in the ten weeks between 11 March and 20 May 2019, formed the 2019 cohort. Patients whose admission was not due to traumatic injuries or who, during their hospital care, were transferred to be managed by the Major Trauma team, were excluded from the analysis. All patients were either added prospectively or retrospectively onto the study’s REDCap database and 25% of the study data were validated independently by two study authors. There were no age restrictions [14].

### Primary outcome

The primary outcome was to quantify and compare the total number of trauma call activations between the two study time periods.

### Secondary outcomes

The secondary outcomes were to quantify and compare differences in Injury Severity Scores (ISS); mechanism of traumatic injury and mortality 30 days from admission. As well as SARS-CoV-2 infection prior to and during admission.

### Exposure definitions

Patients were split into two cohorts: 2019 or 2020 depending on the year of their trauma admission. Age was defined as number of years old on date of admission and categorised into consecutive groups. ISS was split into minor and major severity as previously defined within the literature [16]. Body mass index (BMI) was categorised into obese (> 30) or non-obese (≤ 30) groups. Mechanism of injury was split into nine distinct categories pre-defined by the Major Trauma unit: Blows, Burn, Crush, Fall < 2 m, Fall > 2 m, Shooting, Stabbing, Vehicle Incident/Collision and Other. Frailty was calculated using the Rockwood clinical frailty scale (CFS) [17]. CFS scores were grouped into three sequential categories: Non frail (CFS 1–3), Vulnerable to Mildly frail (CFS 4–5) and Moderate to Severely frail (CFS 6–9). Comorbidity was defined using the Charlson co-morbidity index and grouped into four consecutive categories [18]. SARS-CoV-2 diagnosis was defined as any patient receiving a positive PCR swab result (reverse transcription polymerase chain reaction) or radiologist report of SARS-CoV-2 pneumonitis on CT thorax within 30 days of admission. Socioeconomic status was calculated using each patient’s address postal code from the English Index of Multiple Deprivation (IMD) 2015 and grouped into quintiles with 1 (the most), to 5 (the least) deprived. Ethnicity was determined from the existing demographic data held within hospital records and defined into the categories Asian, Black, Mixed, Other and White.

### Statistical analysis

The data were analysed using Stata V16 (StataCorp, Stata Statistical Software: Release 16, College Station, Texas, USA). Descriptive statistics were used to report the demographics of the two cohorts. Weekly admissions for trauma were calculated and compared for the two time periods to estimate percentage per-week change in admissions. A rolling two weekly average in trauma admission over the 10-week period was compared using the differences in means. Fisher’s exact and Chi squared (*χ*^2^) tests were used to compare categorical variables as appropriate. Univariable and multivariable logistic regression models were used to explore factors that predicted 30-days mortality with the Likelihood ratio test (LRT) used to assess significance. In all analyses, significance was set at the 95% level and *p* < 0.05 was considered significant.

### Ethics and consent

This study was registered with and approved by the local institutional review board as a service evaluation, registration number: 20-177C. Individual patient consent was waived.

## Results

### Demographics

A total of 642 patients were included from the two study periods. There were 405 trauma admissions during the 2019 time period (2019 cohort) and 237 in the 2020 period (2020 cohort), representing a greater than 41% drop in emergency trauma admissions in the latter cohort. In both cohorts, there was a significantly higher proportion of male as compared to female patients, however, there was no statistically significant difference in the male to female ratios between the two cohorts (*χ*^2^ *p* = 0.347). The median age of the 2019 cohort was 40 (IQR 24–59) years and the 2020 cohort 46 (IQR 28–60) years (*p* = 0.050). The 2020 cohort was more moderate to severely frail (*χ*^2^ trend *p* = 0.0015). There was also a statistically significant difference in ethnicity with more injuries identified in the White ethnic group (74.8% in 2019 increased to 85.2% in 2020 cohort) and a drop in the Asian ethnic group from 7.2 to 3.0% (*χ*^2^ *p* = 0.0315). There was no difference in BMI or smoking status between the two cohorts. The patient demographics are summarised in Table 1.

**Table 1:** Demographics and characteristics of cohort by pre and during the COVID-19 era

	2019 (*n* = 405)		2020 (*n* = 237)		*p* value^a^
Age (years)					
0–17	42	10.37%	16	6.75%	0.1572^b^
18–39	154	38.02%	87	36.71%	
40–64	136	33.58%	86	36.29%	
≥ 65	73	18.02%	48	20.25%	
Sex					
Female	120	29.63%	62	26.16%	0.347
Male	285	70.37%	175	73.84%	
ISS					
0—15 (Minor trauma)	309	76.30%	177	74.68%	0.6459
≥ 16 (Major trauma)	96	23.70%	60	25.32%	
BMI (Kg/m^2^)					
≤ 30	385	95.06%	230	97.05%	0.227
> 30	20	4.94%	7	2.95%	
Smoking status					
Non-smoker	357	88.15%	200	84.39%	0.1753
Current smoker	48	11.85%	37	15.61%	
Mechanism of injury					
Blows	9	2.22%	5	2.11%	0.001^c^
Burn	7	1.73%	5	2.11%	
Crush	6	1.48%	3	1.27%	
Fall < 2 m	83	20.49%	63	26.58%	
Fall > 2 m	49	12.10%	54	22.78%	
Other	9	2.22%	7	2.95%	
Shooting	2	0.49%	1	0.42%	
Stabbing	44	10.86%	25	10.55%	
Vehicle incident/collision	196	48.40%	74	31.22%	
Rockwood clinical frailty scale					
Non frail (1–3)	364	89.88%	197	83.12%	0.0015^b^
Vulnerable to Mildly frail (4–5)	29	7.16%	13	5.49%	
Moderate to Severely frail (6–9)	12	2.96%	27	11.39%	
Charlson comorbidity score					
0 (98% 10-years survival)	235	58.02%	134	56.54%	0.1976^b^
1–2 (≥ 90% 10-years survival)	96	23.70%	55	23.21%	
3–4 (> 50% 10-years survival)	52	12.84%	23	9.70%	
≥ 5 (< 25% 10-years survival)	22	5.43%	25	10.55%	
Ethnicity					
Asian	29	7.16%	7	2.95%	0.0315
Black	13	3.21%	6	2.53%	
Mixed	6	1.48%	2	0.84%	
Other	54	13.33%	20	8.44%	
White	303	74.81%	202	85.23%	
COVID-19 diagnosis					
Negative	405	100.00%	233	98.31%	0.018^c^
Positive	0	0.00%	4	1.69%	
30-days mortality					
No	386	95.31%	214	90.30%	0.0132
Yes	19	4.69%	23	9.70%	
SES					
1 (most deprived)	89	21.98%	47	19.83%	0.4032^b^
2	85	20.99%	52	21.94%	
3	79	19.51%	44	18.57%	
4	76	18.77%	41	17.30%	
5 (least deprived)	73	18.02%	52	21.94%	
Missing	3	0.74%	1	0.42%	

*ISS* injury severity score, *BMI* body mass index, *SES* socioeconomic status

^a^Chi-squared test (*χ*^2^)

^b^Chi-squared test (*χ*^2^) for trend

^c^Fisher’s exact test

### Impact of lockdown on trauma calls

**Fig. 1:**
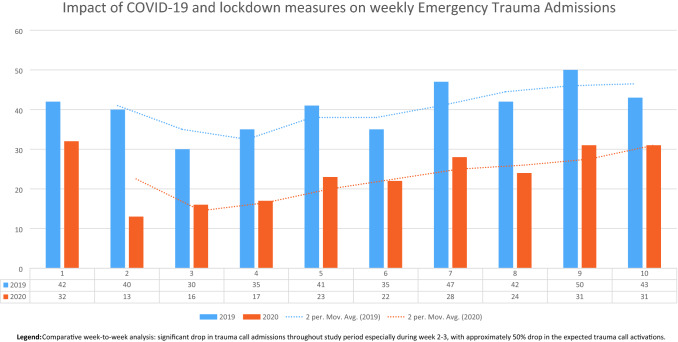
Trend of trauma call admissions (by count and rolling average). Comparative week-to-week analysis: significant drop in trauma call admissions throughout study period especially during week 2–3, with approximately 50% drop in the expected trauma call activations

During the initial phase of the 2020 study period, the total number of trauma call activations fell to its lowest point in week 2, which represented only 32.5% (*n* = 13) of the trauma call activations in the same time period in 2019. However, as the most stringent measures were eased, the proportion of trauma activations increased, rising to 72% (*n* = 32) of the 2019 cohort by the 10th week of the study period. See Fig. 1.

### Mechanism of injury and mortality

Road Traffic collisions (RTCs), in adolescents and falls (< 2 and > 2 m) overall are the commonest causes of trauma admissions in our centre and the UK [19–21]. However, the proportions they contributed differed significantly between the two cohorts (Fisher’s exact *p* = 0.001). In the 2019 period, RTCs were the commonest cause of trauma call activations and represented 48.4% of all trauma admissions, whereas total falls (both falls less than and greater than 2 m) accounted for 32.6%. Contrastingly, in the 2020 cohort, RTCs accounted for 31.2% of trauma call activations, whereas total falls accounted for 49.4%.

Falls from a height above 2 m increased numerically and proportionally between the two cohorts. In 2019, falls of greater than 2 m were responsible for 12.1% of trauma call activations (49/405), and in 2020, they were responsible for 22.8% (54/237). A younger and predominantly male demographic was identified (68.5% of injuries in patients less than 65 years old) in the falls greater than 2 m in the 2020 cohort. Falls from a height below 2 m increased proportionally between the two cohorts. In 2019, falls of less than 2 m were responsible for 20.5% of trauma call activations (83/405), and in 2020, they were responsible 26.6% (63/237). An older and predominantly male demographic was identified (38.1% of injuries in patients greater than or equal to 65 years old) in the falls less than 2 m in the 2020 cohort.

**Fig. 2:**
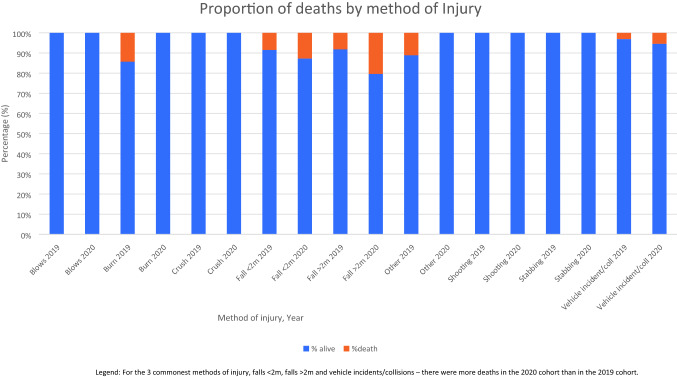
Deaths by Method of injury. For the 3 commonest methods of injury, falls < 2 m, falls > 2 m and vehicle incidents/collisions—there were more deaths in the 2020 cohort than in the 2019 cohort

There were 23 deaths (9.7%) in the 2020 cohort, compared with 19 deaths (4.7%) in the 2019 cohort (*χ*^2^ *p* = 0.0132). For each of the three commonest causes of injuries, there was a proportionally higher risk of death in the 2020 cohort than in the 2019 cohort (*χ*^2^ *p* = 0.0048). See Table 2 and Fig. 2.

**Table 2:** Mortality by method of injury

Mechanism of injury	2019		2020	
	Total	Number of deaths (%)	Total	Number of deaths (%)
Blows	9	0	5	0
Burns	7	0	5	0
Crush	6	1 (14%)	3	0
Fall < 2 m	83	7 (8%)	63	8 (13%)
Falls > 2 m	49	4 (8%)	54	11 (20%)
Other	9	1 (11%)	7	0
Shooting	2	0	1	0
Stabbing	44	0	25	0
Vehicle incidents	196	6 (3%)	74	4 (5%)
Total	405	19 (5%)	237	23 (10%)

### Univariable and multivariable logistical regression of 30-days mortality

In univariable analysis, there was a greater than twofold increased risk of death in patients in the 2020 cohort compared to the 2019 cohort (LRT *p* = 0.0151). Elderly age, frailty, injury severity, co-morbidity and mechanism of injury (falls) were associated with an increased risk of death. A positive SARS-CoV-2 status was also associated with a significantly increased risk of death (LRT *p* = 0.0169) in univariable analysis. However, in the adjusted model, only injury severity (LRT *p* < 0.0001) and frailty (LRT trend *p* = 0.001) were significant associated with an increased risk of mortality. For univariable and multivariable analysis see Table 3.

**Table 3:** Multivariable analysis of 30-days mortality post trauma event

	Unadjusted OR (*n* = 642)	95% CI		*p* value^a^	Adjusted OR (*n* = 547)^c^	95% CI		*p* value^a^
Year								
2019	1.00	(Reference)		0.0151	1.00	(Reference)		0.3168
2020	2.18	1.163	4.100		1.53	0.665	3.540	
Age (years)								
0–17	1.00	(Reference)		< 0.0001^b^	1.00	(Reference)		0.7527^b^
18–39	0.23	0.032	1.700		0.30	0.038	2.398	
40–64	1.74	0.382	7.944		0.71	0.097	5.195	
≥ 65	7.29	1.664	31.952		0.97	0.085	11.179	
Sex								
Female	1.00	(Reference)		0.0807	1.00	(Reference)		0.6492
Male	0.56	0.294	1.060		0.82	0.351	1.918	
ISS								
0–15 (minor trauma)	1.00	(Reference)		< 0.0001	1.00	(Reference)		< 0.0001
≥ 16 (major trauma)	12.28	5.878	25.670		13.42	5.456	33.017	
Mechanism of injury^d^								
Vehicle incident/collision	1.00	(Reference)		0.0066	1.00	(Reference)		0.2511
Blows	1.00	–	–		1.00	–	–	
Burn	2.36	0.277	20.137		0.81	0.032	20.009	
Crush	1.00	–	–		1.00	–	–	
Fall < 2 m	2.98	1.302	6.809		0.75	0.245	2.282	
Fall > 2 m	4.43	1.921	10.223		2.18	0.794	6.007	
Other	1.73	0.208	14.448		2.97	0.267	32.986	
Shooting	1.00	–	–		1.00	–	–	
Stabbing	1.00	–	–		1.00	–	–	
Rockwood clinical frailty scale								
Non frail (1–3)	1.00	(Reference)		< 0.0001^b^	1.00	(Reference)		0.0010^b^
Vulnerable to Mildly frail (4–5)	9.43	4.024	22.084		5.26	1.218	22.745	
Moderate to Severely frail (6–9)	16.89	7.550	37.797		15.69	2.694	91.326	
Charlson comorbidity score								
0 (98% 10-years survival)	1.00	(Reference)		< 0.0001^b^	1.00	(Reference)		0.6552^b^
1–2 (≥ 90% 10-years survival)	4.46	1.722	11.572		2.20	0.515	9.421	
3–4 (> 50% 10-years survival)	7.96	2.923	21.655		1.04	0.132	8.142	
≥ 5 (< 25% 10-years survival)	19.77	7.393	52.885		0.91	0.100	8.324	
SARS-CoV-2 diagnosis								
Negative	1.00	(Reference)		0.0169	1.00	(Reference)		0.1225
Positive	14.95	2.052	108.923		5.83	0.664	51.285	

*SES* socioeconomic status (1—least to 5—most deprived), *ISS* injury severity score

^a^Likelihood ratio test

^b^Likelihood ratio test for trend

^c^Adjusted for all other variables within table

^d^Blows, Crush, Shooting and Stabbing dropped due to predicting failure perfectly, unadjusted analysis *n* = 547

## Discussion

This study demonstrated that behavioural change as a result of the legislation enforcing a lockdown in the UK had a marked effect on the volume and demographic of major trauma presentations. This may be related to the lack of opportunity to engage in activities usually associated with injury, such as; excessive consumption of alcohol, interpersonal violence and RTCs [22, 23]. The scale of change, by as much as 68% reduction in the early phase of the lockdown, is unprecedented and differed significantly from the seasonal variations (which ranges from 10 to 20%) previously reported [5–7]. Profound differences in the demographics of the two matched periods were observed. Patients from the 2020 cohort were older, more co-morbid and frailer in comparison with those from the matched 2019 cohort.

This demographic change was associated with a statistically significant rise in mortality in the 2020 cohort (9.7% compared to 4.7%). This mortality rise was associated with those who are elderly, frail, co-morbid and with a higher ISS. Whilst historically, Major Trauma was associated with young, healthy males with little co-morbidity [24], this study demonstrates that the SARS-CoV-2 pandemic and associated lockdown measures, have accelerated the ‘changing face of trauma’ described by Kehoe et al. [21].

Whilst RTCs were the cause of the majority of admissions in 2019, during the lockdown period in 2020 this was overtaken by falls (both those greater or less than 2 m). There was an increase in the proportion of presentations as a result of falls < 2 m in 2020 compared to 2019. Given the majority of such presentations occur in those aged 65 and over, coupled with the increase in the number of elderly patients presenting in 2020, it suggests that lockdown measures may increase the likelihood of such injuries in this population. Factors which contribute to this may include a reduction in informal care and social support, often provided by family members not living in the same house. In an analysis of care requirements in those aged over 70 years, Evandrou et al. found that 26% of those living alone who required help with one activity of daily living (ADL) prior to the SARS-CoV-2 lockdown were receiving no help during the first 4 weeks of lockdown, whilst 17% of those who required help with two ADLs received no help [25].

This points to a situation in which a notable proportion of elderly people, who are already deemed vulnerable, will struggle with basic tasks on a daily basis, and therefore be at higher risk of falls. This may explain the rise in presentations in elderly, frail patients with falls. In addition, the documented reduction in presentations to General Practice Primary Care services [26] may have resulted in deterioration of underlying health conditions and non-diagnosis and management of new conditions, resulting in falls (often a presentation of an underlying health problem) and trauma admission.

Initial anecdotal evidence from the Italian experience [10] suggested a drop in major trauma admissions, similar to the pattern seen in emergency admissions to medical and surgical specialties [9]. However they pointed to a more severe injury presentation, as measured by ISS [10]. Forrester et al. reported a significant drop in trauma admissions in a matched study from the United States which compared their “shelter at home” lockdown period to a similar time period in 2018 and 2019 [12]. Contrastingly, they did not find any statistically significant differences in demographics, mechanism of injury or severity of injuries. Another study from Spain [11], that included both orthopaedic injuries and major trauma injuries in the same setting, also reported a decrease in RTCs, workplace accidents and a total number of hospital admissions due to trauma after imposition of the Spanish State of Emergency. Similarly, a recent cohort study from South Africa found an overall reduction in emergency trauma admissions, in particular RTCs, but no difference in injury severity during the nation’s lockdown period of April 2020 compared to the previous two consecutive years [27]. Within the UK, a small observational study found a reduction in orthopaedic trauma referrals but no differences in mechanism of injury. [4] Our findings are consistent with the significant reduction in trauma admissions seen in these studies. Importantly, it also points to the change in demographic, method and severity of trauma injuries during the lockdown period. According to the Trauma and Audit Research Network (TARN) study in 2015 [21], the elderly were identified as a vulnerable group who now make up a significant proportion of major trauma admissions. This vulnerable patient group appear to sustain significant injuries even from falls less than 2 m and have a higher risk of mortality. This finding is consistent with other studies which demonstrate a twofold increased risk of mortality in the elderly population compared to younger patients [21, 28].

Overall, this change in demographic and injury characteristics during the lockdown period can be used to inform changes in health care service provision during future regional or national lockdowns. It suggests that whilst trauma services may expect an initial reduction in overall injuries, mainly RTCs, rates of falls particularly in the elderly, may in fact increase, requiring greater input from geriatric, orthopaedic and neurosurgical services. The impact of a positive SARS-CoV-2 diagnosis on patient outcomes cannot be determined by this study, owing to the low number of patients with a positive diagnosis (4/237, 1.69%).

This study was undertaken at a large Major Trauma Centre in the United Kingdom. However, it is a single centre study and therefore patterns of injury and demographics are not necessarily reflected elsewhere in the UK. For instance, RTCs remain the most common cause of trauma admissions at this centre (prior to SARS-CoV-2); however, this is not the case nationally, and in other centres falls < 2 m is the most common cause of trauma admissions [20, 21, 29]. Indeed the lockdown measures causing the elderly to isolate in other UK regions may lead to an increase in falls less than 2 m with a rise in low energy traumas [20]. Additionally, whilst the study database was prospectively maintained the study itself was conducted retrospectively and is therefore subject to potential selection bias.

## Conclusion

During the SARS-CoV-2 pandemic and the associated national lockdown that occurred in the UK between March and May 2020, there was a significant reduction in number of trauma admissions. However, the patient cohort admitted during this period changed: they were older, frailer and more co-morbid, with a higher overall ISS and risk of mortality. These more injured and frail cohort of patients, confirms the importance of prioritising major trauma care throughout this and future pandemics. In addition, resource allocation has to be targeted to support older frailer people, who are at greater risk of falls and its unfavourable consequences.

## Data Availability

Data available on request via corresponding author.
